# Performance of the 2019 ESC/EASD guideline strategy for the screening of silent coronary artery disease in patients with diabetes

**DOI:** 10.1186/s12933-023-01760-4

**Published:** 2023-02-15

**Authors:** Paul Valensi, Narimane Berkane, Sara Pinto, Nicolas Sellier, Michael Soussan, Minh Tuan Nguyen, Emmanuel Cosson

**Affiliations:** 1 Unit of Endocrinology-Diabetology-Nutrition, AP-HP, Jean Verdier Hospital, Paris 13 University, Sorbonne Paris Cité, CRNH-IdF, CINFO, Avenue du 14 Juillet. 93140, Bondy, France; 2 Department of Endocrinology-Diabetology-Nutrition, AP-HP, Avicenne Hospital, Paris 13 University, Sorbonne Paris Cité, CRNH-IdF, CINFO, Bobigny, France; 3 Department of Radiology, AP-HP, Jean Verdier Hospital, Paris 13 University, Sorbonne Paris Cité, Bondy, France; 4 Department of Nuclear Medicine, AP-HP, Avicenne Hospital, Paris 13 University, Sorbonne Paris Cité, Bobigny, France; 5 Unité de Recherche Epidémiologique Nutritionnelle, Paris 13 University, Sorbonne Paris Cité, UMR U557 INSERM/U11125 INRAE/CNAM/Université Paris13, Bobigny, France

**Keywords:** Diabetes, Coronary disease, Silent ischemia, CAC score, Myocardial scintigraphy

## Abstract

**Background:**

The 2019 guidelines for cardiovascular risk stratification by the European Society of Cardiology and European Association for the Study of Diabetes (ESC-EASD) suggested screening for silent coronary disease in very high risk patients with severe target organ damage (TOD) (i.e. peripheral occlusive arterial disease or severe nephropathy) or high coronary artery calcium (CAC) score. This study aimed to test the validity of this strategy.

**Methods:**

In this retrospective study, we included 385 asymptomatic patients with diabetes and no history of coronary disease but with TOD or ≥ 3 risk factors in addition to diabetes. CAC score was measured using computed tomography scan and a stress myocardial scintigraphy was performed to detect silent myocardial ischemia (SMI), with subsequent coronary angiography in those with SMI. Various strategies to select patients to be screened for SMI were tested.

**Results:**

CAC score was ≥ 100 Agatston units (AU) in 175 patients (45.5%). SMI was present in 39 patients (10.1%) and among the 30 patients who underwent angiography, 15 had coronary stenoses and 12 had a revascularization procedure. The most effective strategy consisted in performing myocardial scintigraphy in the 146 patients with severe TOD and, among the 239 other patients without severe TOD, in those with CAC ≥ 100 AU: this strategy provided 82% sensitivity for SMI diagnosis, and identified all the patients with stenoses.

**Conclusion:**

The ESC-EASD guidelines suggesting SMI screening in asymptomatic patients with very high risk assessed by severe TOD or high CAC score appears effective and could identify all the patients with stenoses eligible for revascularization.

## Introduction

The prevalence of diabetes has been increasing worldwide. Diabetes is associated with an increased but heterogeneous risk for developing cardiovascular (CV) disease. Early detection of CV disease is challenging as it may motivate a lot of explorations, the benefits of which have not been well established yet.

A new CV risk stratification was suggested in the guidelines on diabetes, pre-diabetes and cardiovascular disease published in 2019 by the European Society of Cardiology (ESC) in collaboration with the European Association for the Study of Diabetes (EASD) to help practitioners deciding for investigations, setting treatment goals and choosing the most appropriate drugs for their patients with diabetes [1]. This stratification includes well-known risk factors for CV events and emphasizes the role of some CV alterations, which may be considered as risk modifiers. According to these guidelines, very high risk in patients with diabetes includes *(i)* established CV disease or other target organ damage (TOD) including severe nephropathy, left ventricular hypertrophy or diabetic retinopathy; *(ii)* three or more major risk factors or *(iii)* early onset type 1 diabetes of duration > 20 years.

The atherosclerotic burden can also be estimated by calculating the coronary artery calcium (CAC) score on a computed tomography (CT) scan. A high CAC score is associated with a higher risk of CV events and mortality [2–4]. Thus, this score may be considered as a risk modifier in patients with diabetes and no history of CV disease. In addition, some studies have shown an association between high CAC score and silent myocardial ischemia (SMI) [5, 6].

Screening for SMI in asymptomatic patients with diabetes might improve CV prognosis through intensive medical treatment and identification of coronary stenoses eligible for revascularization [1]. However, such a screening is becoming controversial because of the decreasing prevalence of SMI over the last decades [7–9] and because no strong evidence for a benefit could be demonstrated [10].

The ESC-EASD Task Force experts did not recommend routine coronary artery disease (CAD) screening in asymptomatic individuals but asserted that stress testing imaging or CT coronary angiography may be indicated in asymptomatic individuals with a very high CV risk, especially in those with major risk factors such as peripheral occlusive arterial disease (POAD), proteinuria and renal failure as well as in those with high CAC score [1]. However, even if each of these situations is associated with an increased CV risk, the ability of this strategy to detect patients with silent CAD has never been evaluated.

Thus, the aim of this study was to test the validity of this strategy based on major risk factors and CAC score measurement to select asymptomatic patients with diabetes to be screened for SMI, and additionally whether or not this strategy can select patients eligible for coronary revascularization.

## Methods

### Inclusion criteria

Consecutive patients seen between 2010 and 2017 in the diabetes clinic in Jean Verdier Hospital, Bondy, France were retrospectively recruited. The study was observational. Data were extracted from hospital files and were made anonymous. In France, this type of study does not require an approval from an ethics committee/institutional review board or patients’ written informed consent.

We selected individuals with diabetes if they had no symptom or history of CAD, or heart failure, had a normal 12-lead resting electrocardiogram (ECG) and had had both a stress myocardial scintigraphy and a measurement of CAC score. This accounted for about one patient *per* week in our daily practice. Then, we included very high risk patients into four categories:

- Patients with severe TOD, i.e. with POAD (femoral or carotid stenosis ≥ 50% on ultrasound imaging, history of stroke, history of lower limb amputation) or severe nephropathy (urinary albumin excretion rate ≥ 300 mg/day on at least two measurements and/or estimated glomerular filtration rate < 30 ml/min/1.73 m^2^). We did not consider left ventricular hypertrophy since echocardiography was not systematically performed.
- Patients with mild TOD, i.e. with diabetic retinopathy (according to guideline description) or early-stage diabetic nephropathy (urinary albumin excretion rate 30–299 mg/day and/or estimated glomerular filtration rate 30–59 ml/min/1.73 m^2^).
- Patients with three or more major risk factors among age ≥ 50 years in type 2 diabetes or ≥ 35 years in type 1 diabetes, dyslipidemia (total cholesterol > 6.5 mmol/L and/or LDL-cholesterol > 4.1 mmol/L and/or HDL-cholesterol < 0.9 mmol/L and/or triglycerides > 2.3 mmol/L and/or lipid lowering medication), hypertension (blood pressure ≥ 140/90 mmHg or anti-hypertensive treatment), smoking and obesity (body mass index ≥ 30 kg/m^2^).
- Patients with type 1 diabetes with onset before the age of 10 and duration > 20 years.

The study population and further analyses were limited to the patients who could be classified in at least one of these categories.

### Characterization of the population

Clinical examination data were extracted from the patients’ charts, including diabetes complications (retinopathy, nephropathy, peripheral neuropathy, peripheral occlusive arterial disease). Routine lab tests results were also collected: HbA1_c_ (measured with Dimension® technology, Siemens Healthcare Diagnosis Inc., Newark, USA), serum total and HDL-cholesterol as well as triglycerides (enzymatic colorimetry, Hitachi 912, Roche Diagnostics, Meylan, France), serum creatinine (colorimetry, Kone Optima, Thermolab System, Paris La Défense, France), 24 h proteinuria and urinary albumin excretion rate (immunoturbidimetry, Cobas c501, Roche Diagnostics, Meylan, France). LDL-cholesterol was calculated with the Friedewald formula and glomerular filtration rate was estimated (eGFR) using the Modification of Diet in Renal Disease (MDRD) Study equation.

### Stress myocardial scintigraphy

As previously reported [11], patients underwent a dual-isotope rest ^201^thallium/stress ^99m^Tc-sestamibi protocol or a stress/rest protocol using ^99m^Tc-sestamibi. The stress test consisted of an exercise test using a calibrated bicycle ergometer, a pharmacological stress test (dipyridamole injection), or both. ECG stress test was performed when the patient was able to exercise on a bicycle ergometer and was expected to have an interpretable exercise ECG. If the patient was unable to exercise or when the ECG stress test result was indeterminate, a pharmacological stress test was performed. SMI was defined by evidence of an abnormal ECG stress test and/or abnormal myocardial scintigraphy imaging (*i.e.*, defects in at least 3 out of 17 segmental regions).

Patients with SMI were subsequently referred to the cardiologist who decided whether or not coronary angiography should be performed in order to detect coronary stenoses. Coronary stenosis was defined as ≥ 70% narrowing of the luminal diameter in the left anterior descending artery, the circumflex artery, a well-developed marginal vessel or the right coronary artery or ≥ 50% narrowing in the left main coronary artery diameter [11]. Cardiologists decided to undergo a revascularization procedure, consisting in coronary angioplasty with stent or by-pass grafting.

### CAC score

CAC imaging was performed without contrast, using a Revolution HD (General Electric Medical Systems, Milwaukee, Wisconsin). With CT slice thickness of 2.5 mm and defined physical parameters (120 kv, 200 mA, FOV 25.1 cm and 30% ASIR reduction), a total of 48 to 64 slices (clusters of 8 slices by 20 mm section) was obtained starting at the level of carina and proceeding to the level of the diaphragm. Scan time was 350 ms per slice, synchronized to 70% of R-R interval. A calcified lesion was defined as > 3 contiguous pixels with a peak attenuation of at least 130 Hounsfield units. Quantitative calcium scores were calculated according to the method described by Agatston et al. [12].

### Statistical analyses

No data replacement procedure was used for missing data. Continuous variables were expressed as means ± SD. We explored the parameters associated with SMI: continuous variables were compared by one-way ANOVA or the Mann–Whitney’s *U* test as adequate, differences in proportions were tested with the χ^2^ test. Odds ratios (OR) with 95% confidence intervals (95% CI) for the risk of SMI were calculated. Logistic regression was used for multivariable analyses based on models including the factors that were associated with SMI with a p value ≤ 0.10 in univariate analyses.

Several strategies of screening for SMI based on the above-quoted categories and/or CAC scores were tested. The performances of these strategies were analyzed by comparison to the results of screening all very high risk patients that were taken as reference.

Statistical analyses were carried out using SPSS software (SPSS, Chicago, IL). The level of significance for all tests was p < 0.05.

## Results

### Patients’ characteristics

**Fig. 1:**
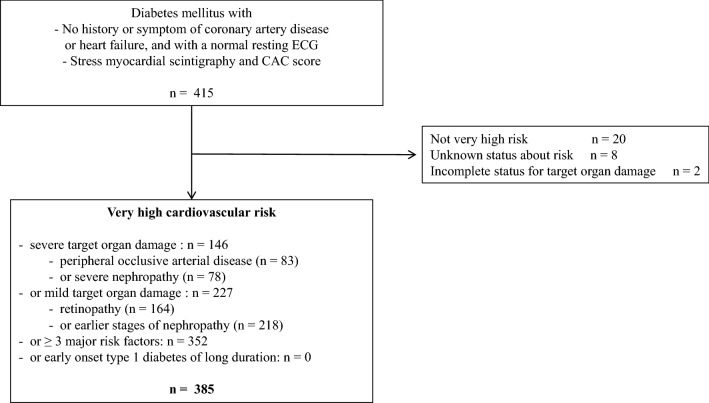
Flow chart of the study. Patients selection according to their very high cardiovascular risk

A total of 415 asymptomatic patients with diabetes could be considered for inclusion. Among them, 28 patients were excluded either because they were not at very high cardiovascular risk (20 patients) or could not be stratified for their risk (8 patients), and 2 more patients were excluded because they did not have adequate TOD evaluation. We finally included 385 patients entering one or more of the four pre-specified categories (flow chart: Fig. 1).

The main characteristics of these 385 patients are shown in Table 1. Mean age was 61.4 ± 9.1 years and mean diabetes duration was 16.2 ± 9.2 years. Most of the population had type 2 diabetes, and 54.3% were on insulin treatment. POAD affected 83 patients (21.7%), and 78 patients (20.5%) had severe nephropathy (macroproteinuria and/or eGFR < 30 ml/min/1.73m^2^). A total of 146 (37.9%) patients had severe TOD (POAD and/or severe nephropathy); 373 (96.9%) patients had mild or severe TOD; 352 (91.4%) had at least 3 risk factors noting that only 12 patients had 3 or more risk factors without any TOD. There was no individual with early onset of long duration type 1 diabetes in our population.

**Table 1:** Patients’ characteristics according to the presence or absence of silent myocardial ischemia

	Total n = 385	SMI + n = 39	SMI − n = 346	p
Clinical characteristics				
Age, years	61.4 ± 9.1	63.5 ± 10.2	61.2 ± 9.0	0.135
Elevated age (%)^a^	356 (92.7)	34 (87.2)	322 (93.3)	0.186
Gender (male/female)	205/180	32/7	173/173	< 0.0001
Diabetes				
Type 1/ Type 2/ other diabetes	27/349/9	2/37/0	25/312/9	0.517
Diabetes duration, years	16.2 ± 9.2	20.3 ± 10.4	15.8 ± 9.0	0.004
HbA1c, %	8.3 ± 1.9	8.2 ± 1.8	8.3 ± 1.9	0.602
Organ damage				
Retinopathy (%)	164 (42.9)	18 (47.4)	146 (42.4)	0.606
eGFR				0.876
30–59 ml/min/1.73m^2^	53 (13.8)	5 (12.8)	48 (14.0)	
< 30 ml/min/1.73m^2^	15 (3.9)	1 (2.6)	14 (4.1)	
Proteinuria				
No	30 (7.9)	2 (5.1)	28 (8.2)	0.772
Microalbuminuria	279 (73.2)	30 (76.9)	249 (72.8)	
Macroproteinuria	72 (18.9)	7 (17.9)	65 (19.0)	
Macroproteinuria or severe renal failure (%)	78 (20.5)	8 (20.5)	70 (20.5)	1.000
Peripheral occlusive arterial disease (%)	83 (21.7)	14 (36.8)	69 (20.1)	0.023
Peripheral neuropathy	273 (72.4)	27 (71.1)	246 (72.6)	0.849
Cardiovascular risk factors				
Family history of premature CAD (%)	50 (13.9)	3 (8.6)	47 (14.5)	0.446
Body mass index, kg/m^2^	31.6 ± 6.3	29.8 ± 5.8	31.8 ± 6.4	0.062
Obesity (%)	216 (57.9)	18 (46.2)	198 (59.3)	0.126
Hypertension (%)	338 (88.7)	37 (97.4)	301 (87.8)	0.102
Lipid parameters				
Total cholesterol, mmol/l	4.2 ± 1.0	4.1 ± 1.1	4.2 ± 1.0	0.722
HDL-cholesterol, mmol/l	1.2 ± 0.4	1.1 ± 0.3	1.2 ± 0.4	0.047
Triglycerides, mmol/l	1.7 ± 0.9	1.7 ± 0.6	1.7 ± 1.0	0.731
LDL-cholesterol, mmol/l	2.2 ± 0.9	2.3 ± 1.0	2.2 ± 0.9	0.545
Dyslipidemia (%)	345 (89.8)	35 (89.7)	310 (89.9)	1.000
Smoking (%)	80 (21.5)	10 (27.8)	70 (20.8)	0.392
Prespecified categories				
Severe TOD	146 (37.9)	20 (51.3)	126 (36.4)	0.082
Any TOD, mild or severe	373 (96.9)	39 (100.0)	334 (96.5)	0.620
At least 3 risk factors (%)^b^	352 (92.1)	36 (92.3)	316 (92.1)	1.000
CAC score				
CAC score (AU)	75.0 [2.6–351.0]	296.0 [73.0–1000.0]	56.7 [1.6–274.9]	< 0.0001
CAC score ≥ 400 AU (%)	86 (22.3)	16 (41.0)	70 (20.2)	0.007
CAC score ≥ 100 AU (%)	175 (45.5)	27 (69.2)	148 (42.8)	0.002

*CAC score (AU)* coronary artery calcium score (Agatston units), *eGFR* estimated glomerular filtration rate, *SMI* silent myocardial ischemia, *TOD* target organ damage

^a^ ≥ 50 years for patients with type 2 diabetes or ≥ 35 years in those with type 1 diabetes

^b^among age, dyslipidemia, hypertension, smoking and obesity

### Cardiologic investigations

Myocardial scintigraphy after ECG stress test (n = 126), dypiridamole injection (n = 134) or both (n = 125) showed SMI in 39 patients (10.1%). A coronary angiography was performed in 30 out of these 39 patients and showed significant coronary stenoses in 15 patients i.e., in half of them. Subsequently, 12 patients had a revascularization procedure (8 coronary angioplasties with stents, 4 coronary artery by-passes).

CAC score was ≥ 400 AU in 86 patients (22.3%) and ≥ 100 AU in 175 patients (45.5%) (Table 1).

### Parameters associated with silent myocardial ischemia

Table 1 shows that parameters associated with the presence of SMI were: male gender (OR 4.6 [95 CI 2.0–10.6]), a longer diabetes duration, a lower HDL-cholesterol level, the presence of POAD (OR 2.3 [1.1–4.7]) and a higher CAC score (CAC score ≥ 400 AU: OR 2.7 [95 CI 1.4–5.5]; CAC ≥ 100 AU: OR 3.0 [95 CI 1.5–6.1]). There was a trend for a lower BMI and a higher prevalence of SMI in patients with severe TOD (20/146 = 13.7%) compared to those without (19/239 = 7.9%) (OR 1.8 [0.9–3.6]; p = 0.082). In addition, there was a trend for a higher prevalence of CS among the patients with severe TOD (9/146 = 6.2%) compared to those without (6/239 = 2.5%) (p = 0.07).

We built three multivariable models (Table 2): *(i)* gender, diabetes duration, body mass index, HDL-cholesterol, POAD and CAC score (model 1); *(ii)* gender, diabetes duration, body mass index, HDL-cholesterol, severe TOD and CAC ≥ 400 AU (model 2); and *(iii)* gender, diabetes duration, body mass index, HDL-cholesterol, severe TOD and CAC ≥ 100 AU (model 3). Male gender, diabetes duration and CAC score level (model 1) or CAC ≥ 100 AU (model 3) were independently associated with SMI.

**Table 2:** Odds ratios and 95% confidence intervals for independent predictors of silent myocardial ischemia in three models of multivariate analyses

Model 1	OR [CI 95]	p
Male gender	3.9 [1.6–9.3]	0.002
Diabetes duration, by 10 years	1.4 [1.0–2.0]	0.04
Body mass index, kg/m^2^		0.336
HDL-cholesterol, mmol/l		0.215
POAD		0.319
CAC score, by 100 AU	1.11 [1.01–1.11]	0.004
Model 2		
Male gender	4.0 [1.7–9.5]	0.002
Diabetes duration, by 10 years	1.5 [1.0–2.1]	0.03
Body mass index, kg/m^2^		0.337
HDL-cholesterol, mmol/l		0.210
Severe TOD		0.731
CAC score ≥ 400 AU	1.9 [0.9–4.0]	0.09
Model 3		
Male gender	4.0 [1.7–9.4]	0.002
Diabetes duration, by 10 years	1.4 [1.0–2.0]	0.04
Body mass index, kg/m^2^		0.266
HDL-cholesterol, mmol/l		0.297
Severe TOD		0.647
CAC score ≥ 100 AU	2.1 [1.0–4.4]	0.054

*AU* agatston unit, *CAC* coronary artery calcium, *POAD* peripheral occlusive arterial disease, *TOD* target organ damage

### Performances of various screening strategies

**Fig. 2:**
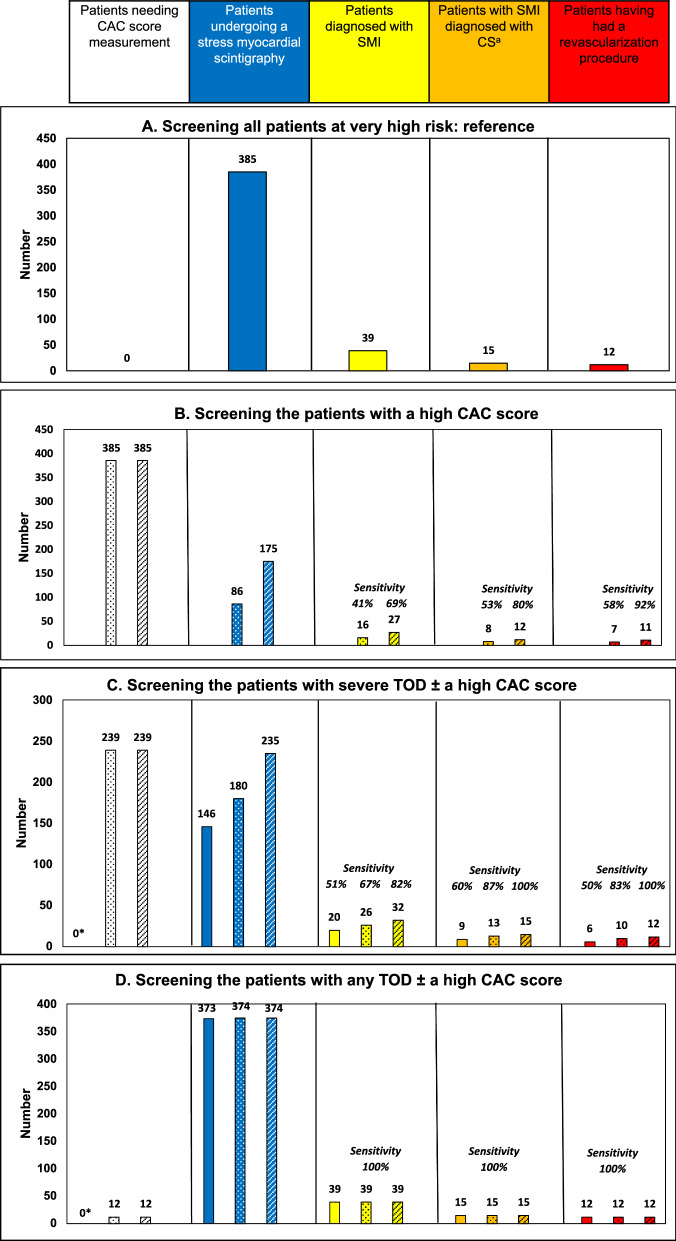
Number of patients undergoing CAC score measurement and stress myocardial scintigraphy; detected with silent myocardial ischemia, coronary stenoses, and who had revascularization procedures according to various screening strategies. Part **A**: Screening all patients at very high risk is considered as the reference strategy. Part **B**: Screening the patients at very high risk and with a high CAC score (dotted bars: CAC score ≥ 400 Agatston units (AU), hatched bars: CAC score ≥ 100 AU). Part **C**: Screening the patients at very high risk and with severe TOD without CAC measurement (solid bars), with severe TOD and CAC score ≥ 400 AU (dotted bars) or ≥ 100 AU (hatched bars). Part **D**: Screening the patients at very high risk and with any TOD (severe or mild) without CAC measurement (solid bars), with any TOD and CAC score ≥ 400 AU (dotted bars) or ≥ 100 AU (hatched bars). The sensitivity to detect the patients with silent myocardial ischemia, coronary stenosis, and who would have had revascularization procedures is shown for strategies **B**–**D**, as compared to screening all patients at very high risk (strategy A*). CAC* score coronary artery calcium score, *CS*: coronary stenosis, *SMI* silent myocardial ischemia, *TOD* target organ damage. ^a^Thirty of the 39 patients with SMI underwent a coronary angiography. *CAC not measured when only the presence of TOD is considered

Figure 2 shows, according to various screening strategies, the number of patients undergoing CAC score measurement and stress myocardial scintigraphy, and the sensitivity of these screening strategies to detect the patients with silent myocardial ischemia, coronary stenosis, and who would have had revascularization procedures. The reference is screening all patients at very high risk (the total cohort).

The strategy could consider screening only the patients with a high CAC score (≥ 400 or ≥ 100 AU). In these cases, 22 and 45% of the patients would have had a myocardial scintigraphy, respectively. Sensitivity for the detection of SMI would be 41 and 69%, respectively. This strategy would offer sensitivity for CS eligible for revascularization procedure of 58% and 92%, respectively.

**Fig. 3:**
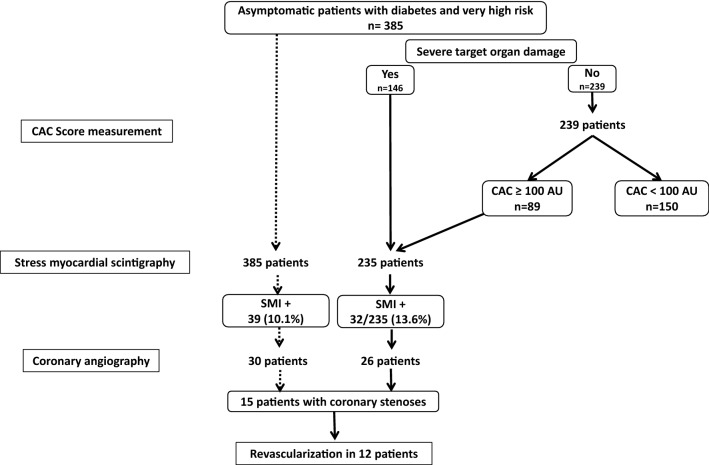
Performances of screening for silent myocardial ischemia patients with severe target organ damage or CAC score ≥ 100 AU (ESC-EASD proposal) compared to screening in all patients with very high cardiovascular risk (reference). *AU* agatston unit, *CAC* coronary artery calcium, *SMI* silent myocardial ischemia

Considering only the 146 patients with severe TOD for screening with myocardial scintigraphy would have missed 19 patients with SMI (sensitivity 51%) and 6 patients with coronary stenoses eligible to revascularization (sensitivity 50%). Would the CAC score measurement be included in the detection strategy in the patients without severe TOD, with myocardial scintigraphy being performed when CAC score is ≥ 400 AU, 47% of the patients would have had a myocardial scintigraphy. The sensitivity of such a strategy would be 67% and 87% for the detection of SMI and CS, respectively. Would a CAC score ≥ 100 AU instead of 400 AU be considered, 61% of the patients would have had a myocardial scintigraphy and the sensitivity for SMI and CS detection would have been 82% and 100%, and all the patients eligible for revascularization would have been identified. To note, the number of coronary angiography indications would also be lowered down to 26 instead of 30. This strategy is illustrated in Fig. 3.

Performing stress myocardial scintigraphy in patients with severe or mild TOD would have lead to test 373 patients and have offered 100% sensitivity to detect SMI and CS. Adding CAC score measurement in the other patients (n = 12) would be useless (no more patients detected).

The performance of SMI assessment in the patients with risk factors and no TOD could not be tested since only 12 patients fulfilled this condition.

## Discussion

Several studies have shown in patients with diabetes that the incidence of acute myocardial infarction [13], death from CV disease [14] and especially from CAD [15] has markedly decreased over the last decades due to a better control of major risk factors. Nevertheless, CV morbidity and mortality remain significantly higher compared to the nondiabetic population. A marked decrease in the prevalence of SMI has also been recently reported in patients with diabetes [9]. However, SMI is more prevalent in some subgroups.

This study is the first one to test the validity of the 2019 ESC/EASD guidelines for CV risk stratification [1], restricting CAD assessment to some very high risk patients. Our results show that among asymptomatic diabetic patients with very high CV risk criteria, a high CAC score was associated with a three-fold increase in SMI risk and was an independent predictor of SMI. Restricting SMI screening to those with severe TOD or high CAC score appears to be a good compromise, selecting all patients with coronary stenoses eligible for revascularization, at a controlled cost.

### Silent myocardial ischemia in diabetes

Myocardial infarction is often silent in patients with diabetes [16, 17]. The prevalence of silent myocardial infarction diagnosed with resting ECG is about 4% and is markedly higher with echocardiography, myocardial scintigraphy or cardiac magnetic resonance [18]. The prevention of silent myocardial infarction is therefore challenging and might be possible with an earlier detection of CAD.

The prevalence of SMI in the diabetic population ranges from 6% [19] to 35% [7]. In our study, using stress myocardial scintigraphy, SMI was diagnosed in about 10% of the patients. This rather low SMI prevalence despite the very high risk patient profile is in line with a recent report [9]. Significant CAD on angiography was reported in 30% [20] to 90% [21] of the patients with SMI; 50% in the current study. Indeed, ischaemia is not always associated with coronary stenoses and may be favoured by functional disorders including endothelial dysfunction, abnormal microcirculation and abnormal coronary reserve [22, 23]; therefore, only few patients with SMI are eligible to revascularization [20, 24]. Noticeably, SMI is predictive of worse CV events [7, 25], especially in the presence of coronary stenoses [20] or with a high CAC score [26], and adds to the prediction above and beyond routine risk assessment [11].

Detection of SMI should promote medical therapy intensification, including an optimal control of risk factors, and may lead to consider coronary revascularization when appropriate [27]. In addition, some CV outcomes trials testing the new glucose-lowering drugs, GLP1-RAs and SGLT2 inhibitors, suggest that these drugs may be beneficial in patients with evidence of SMI and in those with coronary stenoses, thus definitely in very high risk patients [28, 29]. However, this needs to be specifically tested in further studies. Furthermore, screening for SMI does not clearly translate into a reduction in CV events. A recent meta-analysis of randomized controlled trials, focusing on SMI screening and/or treatment, showed that non-invasive screening significantly reduced cardiac events by 27%, a result mostly driven by a decrease in non-fatal myocardial infarction and hospitalization for heart failure [10].

A lot of investigations are still being performed to assess patients for SMI, whereas the potential harms of screening procedures must be carefully evaluated and the cost–benefit ratio of screening has not been definitely established yet. Thus, there is a need to improve SMI screening efficacy. Some markers of SMI have recently been suggested, including resting left ventricular global longitudinal strain [30] and serum oncostatin M, a novel biomarker [31], and need to be tested in further studies. Screening for SMI should probably only be considered in very high risk patients, which would improve the estimation of CV risk and, ultimately, more accurately set therapeutic goals and choose optimal treatment. Indeed, the best strategy to identify silent CAD in patients with diabetes remains unclear. The American Diabetes Association (ADA) guidelines recommend to refrain from screening for silent CAD in asymptomatic individuals with diabetes [32]. The 2019 ESC/EASD guidelines [1] suggested a new CV risk stratification to help selecting patients who should benefit from CV disease and especially SMI screening, in order to set appropriate therapeutic targets and optimize treatments, with a major role for GLP1-RAs and SGLT2 inhibitors. This stratification is primarily based on simple, currently available risk criteria including the age, type and duration of diabetes, the number of associated risk factors and the presence or absence of TOD but also involves some risk modifiers including CAC score that could reclassify more accurately the CV risk. According to these guidelines, screening for SMI may be indicated in the very high risk patients with severe TOD and/or high CAC score.

In our study, we included asymptomatic patients with diabetes and very high CV risk criteria according to the ESC/EASD guidelines [1]. Male gender, a long duration of diabetes, low HDL-cholesterol level and the presence of POAD were associated with a high risk of SMI, in agreement with previous publications. Indeed, diabetic retinopathy [33], nephropathy [19, 34], cardiac autonomic neuropathy [35] and POAD [20, 33] have been shown to be associated with a higher prevalence of SMI. Chronic kidney disease is an independent risk factor for multi-vessel CAD [36, 37], and screening for CAD is important in the preoperative evaluation of kidney transplant candidates [38]. Similarly, POAD is a strong predictor of CAD [39]. Interestingly, in our study, the prevalence of SMI was higher in patients with severe TOD (POAD or severe nephropathy) compared to those without. This finding is supportive of the role of this very high risk component as defined in the ESC/EASD guidelines in selecting patients for SMI screening.

### Role of CAC score in the detection of silent myocardial ischemia

CAC score is a safe, rapid and inexpensive method to assess the volume of coronary calcifications. It is assumed that each calcification equals an atherosclerotic plaque. Among asymptomatic patients with diabetes, the prevalence of elevated CAC score is at or above 20%, that is higher than in the nondiabetic population [2, 40]. The predictive value for mortality of an elevated CAC score is increased in patients with diabetes compared to nondiabetic individuals [2]. CAC score was shown to improve CV risk stratification on top of traditional risk factors [2, 40, 41], and to predict mortality in addition to scintigraphy both in symptomatic and asymptomatic patients [26, 42]. CAC score is considered in the ADA and ESC/EASD guidelines as a risk modifier [1, 32]. In addition, a meta-analysis has reported a quantitative relationship between CAC score level and the likelihood of SMI diagnosis during stress scintigraphy [5]. In our study, CAC score was ≥ 100 AU in 45% of our patients, and high CAC scores were associated with a higher risk of SMI. Interestingly, multivariable analyses showed that CAC ≥ 100 AU – but not POAD or severe TOD—was independently associated with SMI.

We evaluated various strategies to identify the patients with a high likelihood of SMI: performing a stress myocardial scintigraphy in patients with either severe TOD only, or high CAC score only, or in patients with severe TOD or high CAC score in the absence of severe TOD, and finally in those with any TOD (mild or severe). When compared to stress scintigraphy in the overall population (reference data), the third strategy led to perform CAC score measurements in 62% of the total population with a marked reduction (by 39 or 53% when considering the thresholds of 100 or 400 AU for CAC score, respectively) in the number of scintigraphies and a reduction in the number of coronary angiographies. This strategy, using the threshold of 100 AU for CAC score, identified all the patients with significant stenoses including all those eligible for coronary revascularization. Thus, this strategy appeared as the most effective. The three-risk factors criterion, another component of the very high risk profile according to ESC/EASD guidelines, was present in more than 90% of our study population, suggesting that this criterion does not allow an effective selection of patients who should be screened for SMI.

Thus, those results emphasize the role of CAC as a useful marker of SMI in patients with diabetes but no evidence of TOD. Moreover, in a randomized study including patients with no history of CV disease, the patients who were randomized to perform CAC measurement had better subsequent control of their risk factors compared to patients who did not have CAC score measurement; and CAC magnitude was reported to identify patients most likely to benefit from statins in primary CV prevention [43]. CAC score may help practitioners and patients in decision making and encourage the initiation and continuation of preventive therapies. In this respect, it should be emphasized that our strategy including CAC measurement in patients with no evidence of TOD, detected all the patients with significant stenoses eligible for coronary revascularization. Using CAC imaging and secondarily stress scintigraphy may improve diagnostic performance and appear to be a cost-effective strategy, as previously suggested [6, 44]. However, whether or not the detection of silent CAD using CAC score measurement may improve clinical outcomes remains to be shown in specifically designed prospective studies.

### Limitations and strengths

Our study has some limitations. First, the study was retrospective and the results need to be confirmed in a prospective study including a broader population of very high risk patients with diabetes. Second, the participants were recruited in one hospital center, and that could limit the generalizability of our results. However, the study population was reasonably large and relatively homogeneous since all the patients were hospitalized in the same diabetes center and were at very high CV risk according to the ESC/EASD guidelines but with no history or symptoms of CV disease. Regarding to the type of diabetes, the large majority of our patients had type 2 diabetes, which precludes a definitive conclusion for patients with type 1 diabetes. Third, left ventricular hypertrophy, one of the TOD reported in guidelines, could not be considered as echocardiography was not always available. Therefore, the study might have missed some patients that the guidelines define as very high risk. Nevertheless, information about all the other TODs was available. Fourth, the cross-sectional design of our study prevents us from evaluating the impact of the tested strategies on CV outcomes.

Nevertheless, our study has major strengths, as the same protocol was applied to the total population, stress scintigraphy was always performed in the same nuclear medicine department and CAC score was measured in a unique radiology center.

## Conclusion

Among asymptomatic patients with diabetes and a very high CV risk profile, as defined by the 2019 ESC/EASD guidelines, our results suggest that SMI screening restricted to those with severe TOD or high CAC score—as suggested in guidelines—provides good sensitivity and leads to the identification of most patients with coronary stenoses. When using a CAC score threshold ≥ 100 AU, all the patients eligible to revascularization were identified. This strategy seems to be a good cost-effective compromise. In clinical practice, in patients with type 2 diabetes and very high CV risk, SMI assessment could be a two-step strategy: first, identify the patients with severe TOD and measure CAC score in those without severe TOD, and second, perform a stress imaging test in the presence of severe TOD or high CAC score.

## Data Availability

The datasets generated during and/or analysed during the current study are available from the corresponding author on reasonable request.

## References

[1] Cosentino F, Grant PJ, Aboyans V (2020). ESC scientific document group. 2019 ESC guidelines on diabetes, pre-diabetes, and cardiovascular diseases developed in collaboration with the EASD. Eur Heart J..

[2] Raggi P, Shaw LJ, Berman DS, Callister TQ (2004). Prognostic value of coronary artery calcium screening in subjects with and without diabetes. J Am Coll Cardiol.

[3] Greenland P, Blaha MJ, Budoff MJ, Erbel R, Watson KE (2018). Coronary calcium score and cardiovascular risk. J Am Coll Cardiol.

[4] Kramer CK, Zinman B, Gross JL (2013). Coronary artery calcium score prediction of all-cause mortality and cardiovascular events in people with type 2 diabetes: systematic review and meta-analysis. BMJ.

[5] Bavishi C, Argulian E, Chatterjee S, Rozanski A (2016). CACS and the frequency of stress-induced myocardial ischemia during MPI: a meta-analysis. JACC Cardiovasc Imaging.

[6] Anand DV, Lim E, Hopkins D (2006). Risk stratification in uncomplicated type 2 diabetes: prospective evaluation of the combined use of coronary artery calcium imaging and selective myocardial perfusion scintigraphy. Eur Heart J.

[7] Valensi P, Pariès J, Brulport-Cerisier V (2005). Predictive value of silent myocardial ischemia for cardiac events in diabetic patients: influence of age in a French multicenter study. Diabetes Care.

[8] Nguyen MT, Pham I, Valensi P (2014). Flow-mediated-paradoxical vasoconstriction is independently associated with asymptomatic myocardial ischemia and coronary artery disease in type 2 diabetic patients. Cardiovasc Diabetol.

[9] Sultan A, Perriard F, Macioce V (2017). Evolution of silent myocardial ischaemia prevalence and cardiovascular disease risk factor management in type 2 diabetes over a 10-year period: an observational study. Diabet Med.

[10] Clerc OF, Fuchs TA, Stehli J (2018). Non-invasive screening for coronary artery disease in asymptomatic diabetic patients: a systematic review and meta-analysis of randomised controlled trials. Eur Heart J Cardiovasc Imaging.

[11] Cosson E, Nguyen MT, Chanu B (2011). Cardiovascular risk prediction is improved by adding asymptomatic coronary status to routine risk assessment in type 2 diabetic patients. Diabetes Care.

[12] Agatston AS, Janowitz WR, Hildner FJ, Zusmer NR, Viamonte M, Detrano R (1990). Quantification of coronary artery calcium using ultrafast computed tomography. JACC.

[13] Gregg EW, Li Y, Wang J (2014). Changes in diabetes-related complications in the United States, 1990–2010. N Engl J Med.

[14] Rawshani A, Rawshani A, Franzén S (2017). Mortality and cardiovascular disease in type 1 and type 2 diabetes. N Engl J Med.

[15] Dale AC, Vatten LJ, Nilsen TI, Midthjell K, Wiseth R (2008). Secular decline in mortality from coronary heart disease in adults with diabetes mellitus: cohort study. BMJ.

[16] Pride YB, Piccirillo BJ, Gibson CM (2013). Prevalence, consequences, and implications for clinical trials of unrecognized myocardial infarction. Am J Cardiol.

[17] Rangé G, Saint Etienne C, Marcollet P (2019). Factors associated with delay in transfer of patients with ST-segment elevation myocardial infarction from first medical contact to catheterization laboratory: lessons from CRAC, a French prospective multicentre registry. Arch Cardiovasc Dis.

[18] Valensi P, Lorgis L, Cottin Y (2011). Prevalence, incidence, predictive factors and prognosis of silent myocardial infarction: a review of the literature. Arch Cardiovasc Dis.

[19] Milan Study on Atherosclerosis and Diabetes (MiSAD) Group (1997). Prevalence of unrecognized silent myocardial ischemia and its association with atherosclerotic risk factors in noninsulin-dependent diabetes mellitus. Am J Cardiol.

[20] Cosson E, Paycha F, Paries J (2004). Detecting silent coronary stenoses and stratifying cardiac risk in patients with diabetes: ECG stress test or exercise myocardial scintigraphy?. Diabet Med.

[21] Sejil S, Janand-Delenne B, Avierinos JF (2006). Six-year follow-up of a cohort of 203 patients with diabetes after screening for silent myocardial ischaemia. Diabet Med.

[22] Nitenberg A, Ledoux S, Valensi P, Sachs R, Attali JR, Antony I (2001). Impairment of coronary microvascular dilation in response to cold pressor–induced sympathetic stimulation in type 2 diabetic patients with abnormal stress thallium imaging. Diabetes.

[23] Nitenberg A, Valensi P, Sachs R, Dali M, Aptecar E, Attali JR (1993). Impairment of coronary vascular reserve and ACh-induced coronary vasodilation in diabetic patients with angiographically normal coronary arteries and normal left ventricular systolic function. Diabetes.

[24] Muhlestein JB, Lappé DL, Lima JA (2014). Effect of screening for coronary artery disease using CT angiography on mortality and cardiac events in high-risk patients with diabetes: the FACTOR-64 randomized clinical trial. JAMA.

[25] Young LH, Wackers FJ, Chyun DA (2009). DIAD Investigators. Cardiac outcomes after screening for asymptomatic coronary artery disease in patients with type 2 diabetes: the DIAD study: a randomized controlled trial. JAMA..

[26] Chang SM, Nabi F, Xu J (2009). The coronary artery calcium score and stress myocardial perfusion imaging provide independent and complementary prediction of cardiac risk. J Am Coll Cardiol.

[27] Valensi P, Cosson E (2010). It is not yet the time to stop screening diabetic patients for silent myocardial ischaemia. Diabetes Metab.

[28] Valensi P, Prévost G (2020). CVOTs: what did the endocrinologist learn. Diabetes Res Clin Pract.

[29] Buse JB, Wexler DJ, Tsapas A (2020). Management of hyperglycaemia in type 2 diabetes, 2018 a consensus report by the american diabetes association (ADA) and the European association for the study of diabetes (EASD). Diabetologia.

[30] Albenque G, Rusinaru D, Bellaiche M, Di Lena C, Gabrion P, Delpierre Q, Malaquin D, Tribouilloy C, Bohbot Y (2022). Resting left ventricular global longitudinal strain to identify silent myocardial ischemia in asymptomatic patients with diabetes mellitus. J Am Soc Echocardiogr.

[31] Ikeda S, Sato K, Takeda M, Miki K, Aizawa K, Takada T, Fukuda K, Shiba N (2021). Oncostatin M is a novel biomarker for coronary artery disease—a possibility as a screening tool of silent myocardial ischemia for diabetes mellitus. Int J Cardiol Heart Vasc..

[32] American Diabetes Association (2021). 10 cardiovascular disease and risk management: standards of medical care in diabetes-2021. Diabetes Care.

[33] Janand-Delenne B, Savin B, Habib G, Bory M, Vague P, Lassmann-Vague V (1999). Silent myocardial ischemia in patients with diabetes: who to screen. Diabetes Care.

[34] Gazzaruso C, Giordanetti S, De Amici E (2004). Relationship between erectile dysfunction and silent myocardial ischemia in apparently uncomplicated type 2 diabetic patients. Circulation.

[35] Vinik AI, Maser RE, Mitchell BD, Freeman R (2003). Diabetic autonomic neuropathy. Diabetes Care.

[36] Ninomiya T, Perkovic V, de Galan BE (2009). ADVANCE collaborative group. albuminuria and kidney function independently predict cardiovascular and renal outcomes in diabetes. J Am Soc Nephrol..

[37] Hakeem A, Bhatti S, Karmali KN (2010). Renal function and risk stratification of diabetic and nondiabetic patients undergoing evaluation for coronary artery disease. JACC Cardiovasc Imaging.

[38] Cai Q, Mukku VK, Ahmad M (2013). Coronary artery disease in patients with chronic kidney disease: a clinical update. Curr Cardiol Rev.

[39] Reaven PD, Sacks J (2005). Investigators for the VADT coronary artery and abdominal aortic calcification are associated with cardiovascular disease in type 2 diabetes. Diabetologia..

[40] Malik S, Zhao Y, Budoff M (2017). Coronary artery calcium score for long-term risk classification in individuals with type 2 diabetes and metabolic syndrome from the multi-ethnic study of atherosclerosis. JAMA Cardiol.

[41] Budoff MJ, Shaw LJ, Liu ST (2007). Long-term prognosis associated with coronary calcification: observations from a registry of 25,253 patients. J Am Coll Cardiol.

[42] Ramakrishna G, Miller TD, Breen JF, Araoz PA, Hodge DO, Gibbons RJ (2007). Relationship and prognostic value of coronary artery calcification by electron beam computed tomography to stress-induced ischemia by single photon emission computed tomography. Am Heart J.

[43] Rozanski A, Gransar H, Shaw LJ (2011). Impact of coronary artery calcium scanning on coronary risk factors and downstream testing the EISNER (Early Identification of subclinical atherosclerosis by noninvasive imaging research) prospective randomized trial. J Am Coll Cardiol.

[44] Lim E, Lahiri A (2007). The importance of the link between coronary artery calcification and myocardial ischemia: a developing argument. J Nucl Cardiol.

